# Incidence, Management, and Prevention of Gynecomastia and Breast Pain in Patients with Prostate Cancer Undergoing Antiandrogen Therapy: A Systematic Review and Meta-analysis of Randomized Controlled Trials

**DOI:** 10.1016/j.euros.2025.01.001

**Published:** 2025-01-27

**Authors:** Ichiro Tsuboi, Robert J. Schulz, Ekaterina Laukhtina, Koichiro Wada, Pierre I. Karakiewicz, Motoo Araki, Shahrokh F. Shariat

**Affiliations:** a Department of Urology, Comprehensive Cancer Center, Medical University of Vienna, Vienna, Austria; b Department of Urology, Shimane University Faculty of Medicine, Shimane, Japan; c Department of Urology, Okayama University Graduate School of Medicine, Dentistry and Pharmaceutical Sciences, Okayama, Japan; d Department of Urology, University Medical Center Hamburg-Eppendorf, Hamburg, Germany; e Cancer Prognostics and Health Outcomes Unit, University of Montreal Health Centre, Montreal, Quebec, Canada; f Department of Urology, University of Texas Southwestern Medical Center, Dallas, TX, USA; g Department of Urology, Weill Cornell Medical College, New York, NY, USA; h Department of Urology, Second Faculty of Medicine, Charles University, Prague, Czech Republic; i Division of Urology, Department of Special Surgery, The University of Jordan, Amman, Jordan; j Karl Landsteiner Institute of Urology and Andrology, Vienna, Austria; k Department of Urology, Semmelweis University, Budapest, Hungary; l Research Center for Evidence Medicine, Urology Department, Tabriz University of Medical Sciences, Tabriz, Iran

**Keywords:** Antiandrogen therapy, Androgen deprivation therapy, Androgen receptor pathway inhibitors, Breast pain, Gynecomastia

## Abstract

**Background and objective:**

In patients with prostate cancer treated with antiandrogen monotherapy, gynecomastia and breast pain are relatively common. In the setting of androgen receptor pathway inhibitors (ARPIs), the incidence of these adverse events (AEs) remains unclear. In addition, the effect of prophylactic treatment on gynecomastia remains uncertain. We aimed to evaluate the incidence of gynecomastia and breast pain in prostate cancer patients treated with ARPIs compared with androgen deprivation therapy (ADT) and the effect of prophylactic treatment for these AEs due to antiandrogen therapy.

**Methods:**

In June 2024, we queried four databases—PubMed, Scopus, Web of Science, and Embase—for randomized controlled trials (RCTs) investigating prostate cancer treatments involving antiandrogen therapy. The endpoints of interest were the incidence of these AEs due to ARPIs and the effect of prophylactic treatment for these.

**Key findings and limitations:**

Eighteen RCTs, comprising 5036 patients, were included in the systematic review and meta-analysis. ARPIs included enzalutamide, darolutamide, and apalutamide. The results indicated that patients who received ARPI monotherapy had a significantly higher incidence of gynecomastia than those who received ADT monotherapy (risk ratio [RR]: 5.19, 95% confidence interval [CI]: 3.58–7.51, *p* < 0.001). There was no significant difference in the incidence of gynecomastia between ARPI plus ADT therapy and ADT monotherapy (RR: 1.27, 95% CI: 0.84–1.93, *p* = 0.2). Prophylactic tamoxifen or radiotherapy reduced significantly the incidence of gynecomastia and breast pain caused by bicalutamide monotherapy.

**Conclusions and clinical implications:**

We found that ARPI monotherapy increases the incidence of these AEs significantly compared with ADT. In contrast, ARPI plus ADT therapy did not result in a higher incidence of AEs. The use of either tamoxifen or radiotherapy was effective in reducing the incidence of these AEs due to bicalutamide monotherapy. These prophylactic treatments could reduce the incidence of AEs due to ARPI monotherapy. However, further studies are needed to clarify their efficacy.

**Patient summary:**

Although androgen deprivation therapy (ADT) improves overall survival in patients with prostate cancer, it is associated with several complications. Androgen receptor pathway inhibitor (ARPI) monotherapy has emerged as a promising strategy for improving oncological outcomes in these patients. However, ARPI monotherapy increases gynecomastia and breast pain in prostate cancer patients compared with ADT, while ARPI plus ADT did not result in a higher incidence of adverse events.

## Take Home Message

Androgen receptor pathway inhibitor (ARPI) monotherapy increases gynecomastia and breast pain in prostate cancer patients compared with androgen deprivation therapy (ADT), while ARPI plus ADT does not significantly differ from ADT alone.

## 1 Introduction

While androgen deprivation therapy (ADT) improves overall survival in patients with prostate cancer (PCa), it significantly impacts quality of life by contributing to skeletal complications, metabolic syndrome, fatigue, hot flashes, and sexual dysfunction [1]. In recent years, several studies [2], [3], [4], [5], [6], [7] have investigated the effect of androgen receptor pathway inhibitors (ARPIs), a next-generation drug intended to block the interaction between androgens and their receptors, with or without ADT, compared with ADT monotherapy at various stages of PCa. ARPI therapy, with or without ADT, has emerged as a promising strategy for improving oncological outcomes. ARPI monotherapy has been shown to reduce the incidence of hot flashes compared with ADT. However, this approach is likely to lead to a higher incidence of gynecomastia [2], [3].

Gynecomastia and breast pain occurred in up to 85% of patients receiving high-dose bicalutamide monotherapy, while the incidence decreased significantly to 20% in those receiving combined androgen blockade [1], [8]. Gynecomastia results from an imbalance between the effects of free estrogen and free androgen in the breast tissue, and this imbalance may be caused by several different mechanisms [9]. Gynecomastia should be differentiated from pseudogynecomastia, which can present as breast fat, breast cancer, and metastatic tumors. The distinction between gynecomastia and pseudogynecomastia is determined through a physical examination. Gynecomastia is characterized by the presence of a palpable, rubbery, or firm mass of tissue around the nipple-areolar complex [10]. Although gynecomastia is relatively prevalent, treatment for gynecomastia is considerably less highlighted than treatment for other adverse events (AEs). Previous studies [11], [12], [13] revealed that either prophylactic tamoxifen administration or prophylactic radiotherapy (RT) reduced the incidence of gynecomastia and breast pain caused by bicalutamide monotherapy (150 mg/d) compared with no prophylactic treatment.

We conducted this systematic review and meta-analysis to clarify the incidence of gynecomastia and breast pain caused by ARPIs in comparison with those caused by ADT and the effect of prophylactic treatment for gynecomastia and breast pain due to antiandrogen therapy.

## 2 Methods

We registered the study in the International Prospective Register of Systematic Reviews (PROSPERO: registration number: CRD42024550517). This systematic review and meta-analysis was conducted in accordance with the Preferred Reporting Items for Systematic Reviews and Meta-analyses (PRISMA) statement (PRISMA 2020 checklist, and Supplementary Tables 1 and 2).

### 2.1 Search strategy

In June 2024, Embase, PubMed, Scopus, and Web of Science databases were searched to identify studies investigating the incidence of gynecomastia or breast pain due to ARPI therapy, and the effect of prophylactic treatment for gynecomastia or breast pain caused by antiandrogen therapy in patients with PCa. The search terms included the following: “androgen deprivation therapy,” “androgen receptor signaling inhibitors,” “prostate cancer,” and “randomized controlled trial.” The detailed search strategy for each database is provided in the Supplementary material. In addition, we searched the reference lists of the articles to identify additional studies of interest. Two authors independently performed an initial screening based on the titles and abstracts, and noted the reason for exclusion of ineligible reports. Full texts were retrieved and evaluated for eligibility. Disagreements were resolved by consensus among the authors.

### 2.2 Inclusion and exclusion criteria

We used the population, interventions, comparator, outcomes, and study design (PICOS) framework to define the eligibility criteria (Supplementary Table 3) [14]. We included studies that evaluated the incidence rate of gynecomastia and breast pain due to ARPI therapy and the efficacy of prophylactic treatment for gynecomastia and breast pain due to antiandrogen therapy in patients with PCa (population/interventions). We compared these patients with those who received ADT or who underwent antiandrogen therapy without prophylactic therapy. The primary outcomes were gynecomastia and breast pain due to antiandrogen therapy (control/outcomes). We included only randomized controlled trials (RCTs; study design). We excluded studies that lacked original patient data, as well as reviews, letters, editorials, authors’ responses, and case reports. We also excluded retrospective studies. When encountering duplicate cohorts, study quality was determined by two authors based on criteria such as sample size and follow-up duration.

### 2.3 Data extraction

Two authors independently extracted data on baseline study and patients’ characteristics. From each study, we gathered the following essential details: the first author's name, year of publication, country, study period, types and dosages of prophylactic treatment for gynecomastia and breast pain, types and dosages of ARPI therapy or antiandrogen therapy, inclusion and exclusion criteria, number of participants, median duration of follow-up, median age of patients, and incidence of gynecomastia and breast pain. When required outcomes were not available directly in the text, Kaplan-Meier curves and bar graphs were digitized using WebPlotDigitizer software (version 4.6) to obtain survival estimates along with 95% confidence intervals (CIs) [15], [16]. All discrepancies were resolved by consensus among the authors.

### 2.4 Quality assessment and risk of bias

Study quality and risk of bias were evaluated using the Risk-of-Bias version 2 (RoB2) tool, as described in the *Cochrane Handbook for Systematic Reviews of Interventions* [17]. The RoB2 assessment of each study was performed by two authors independently.

### 2.5 Statistical analysis

All statistical analyses were performed using R version 4.2.2 (meta, 2023; R Foundation for Statistical Computing, Vienna, Austria). A random-effect model was conducted to synthesize the outcomes across included studies. Statistical significance was defined as *p* < 0.05. To evaluate the incidence of gynecomastia and breast pain due to ARPI therapy, and the efficacy of prophylactic treatment for gynecomastia and breast pain due to antiandrogen therapy, we generated and analyzed forest plots with risk ratios (RRs) and 95% CIs. Subgroup analyses were performed according to the types of prophylactic treatment. Cochrane’s Q test and the *I^2^* test were used to evaluate the heterogeneity. Significant heterogeneity was indicated by a *p* value of <0.05 in the Cochran’s Q tests and *I^2^* statistics >50%. When significant heterogeneity was observed, we attempted to investigate the causes of heterogeneity [18].

## 3 Results

### 3.1 Study selection and characteristics

**Fig. 1:**
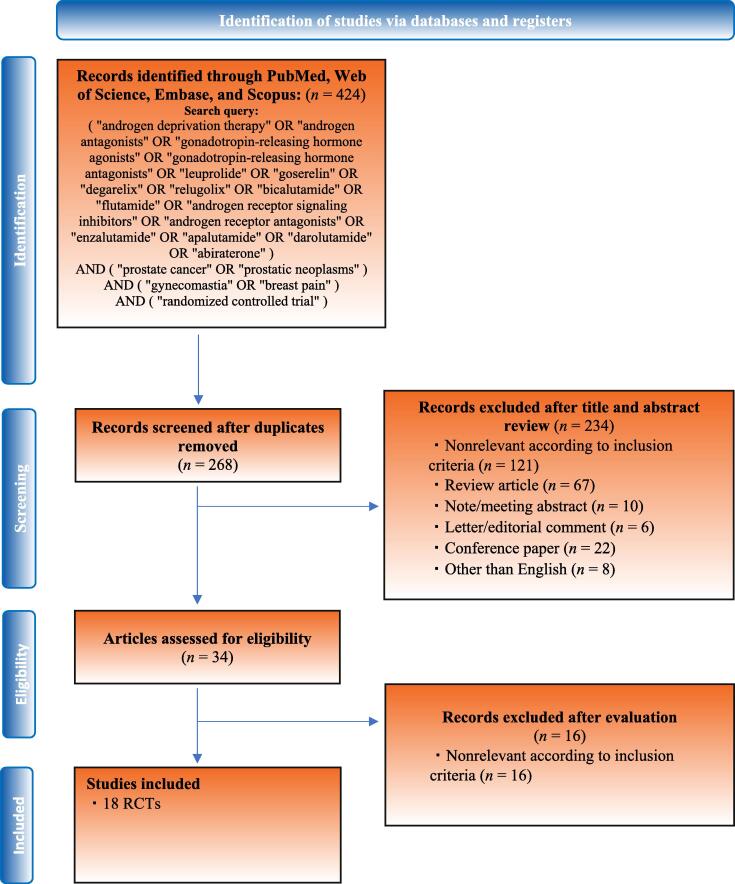
PRISMA flowchart, detailing the article selection process. PRISMA = Preferred Reporting Items for Systematic Reviews and Meta-analyses; RCT = randomized controlled trial.

Our initial search identified 424 records. After the removal of duplicates, 268 records remained for the screening of titles and abstracts, resulting in 234 articles being excluded (Fig. 1). According to our inclusion criteria, we identified 18 RCTs [2], [3], [4], [5], [6], [7], [11], [12], [13], [19], [20], [21], [22], [23], [24], [25], [26], [27] comprising 5773 eligible patients for meta-analyses. Nine RCTs [2], [3], [4], [5], [6], [7], [19], [20], [21], comprising 4578 patients, reported the incidence of gynecomastia and breast pain due to ARPI therapy compared with the incidence of these symptoms due to antiandrogen therapy without ARPIs. Two studies [2], [6] used darolutamide, five studies [3], [4], [5], [7], [21] examined enzalutamide, and two studies [19], [20] used apalutamide. Nine studies [11], [12], [13], [22], [23], [24], [25], [26], [27], comprising 1195 patients, reported a comparison of the incidence of gynecomastia and breast pain between patients who received prophylactic treatment and those who did not. Of the nine studies, eight [11], [12], [13], [22], [23], [24], [25], [26] used bicalutamide monotherapy (150 mg/d) as antiandrogen therapy. Six [11], [13], [22], [23], [24], [25] and four [12], [24], [26], [27] studies investigated the effect of tamoxifen and RT, respectively, as prophylactic treatment for gynecomastia. Details of these treatments are summarized in Table 1.

**Table 1:** Characteristics of included studies

Author (year)	Trial number or name	Period	Treatment	Control	Inclusion criteria of patients	No. of patients Total Treatment Control	Median Age (yr)	Median follow-up duration (mo)	Any grade of gynecomastia, *n* (%)	Any grade of breast pain, *n* (%)
*Incidence of gynecomastia or breast pain*										
Tombal et al (2024) [2]	EORTC-GUCG 1532/NCT02972060	2017–2023	Darolutamide 1200 mg/d monotherapy	ADT	HSPC	61 T:32 C:29	T: 72 C: 74	T: 22 C: 25	T: 11 (34) C: 0 (0)	T: 2 (0.6) C: 0 (0)
Tran et al (2023) [3]	The SALV-ENZA trial/NCT02203695	2015–2020	Enzalutamide 160 mg/d monotherapy for 6 mo	Placebo + salvage RT	BCR	86 T: 43 C: 43	T: 69 C: 66	34	NA	T: 11 (26) C: 0 (0)
Sweeney et al (2023) [4]	ENZAMET/NCT02446405	2014–2017	Enzalutamide 160 mg/d + ADT	Nonsteroidal antiandrogen + ADT	mHSPC	1125 T: 563 C: 562	T: 69 C: 69	68	T: 51 (9) C: 35 (6)	T: 9 (1.6) C: 8 (1.4)
Freedland et al (2023) [5]	EMBARK/NCT02319837	2015–2018	Enzalutamide 160 mg/d + ADT	ADT	BCR	707 T: 353 C: 354	T: 69 C: 70	60.7	T: 29 (8) C: 32 (9)	T: 16 (5) C: 8 (2)
			Enzalutamide 160 mg/d monotherapy	ADT	BCR	708 T: 354 C: 354	T: 69 C: 70	60.7	T: 159 (45) C: 32 (9)	T: 104 (30) C: 8 (2)
Smith et al (2022) [6]	ARASENS/NCT02799602	2016–2018	Darolutamide 1200 mg/d + ADT + DTX	ADT + DTX	mHSPC	1302 T: 652 C: 650	67	44	T: 21 (3) C: 10 (1.5)	NA
Shore et al (2022) [7]	ENACT study/NCT02799745	2016–2020	Enzalutamide 160 mg/d monotherapy for 1 yr	Active surveillance	Localized PCa	227 T: 114 C: 113	T: 65 C: 67	16	T: 41 (36) C: 2 (1.8)	T: 70 (61) C: 1 (0.9)
Aggarwal et al (2022) [19]	NCT01790126	2013–2019	Apalutamide 240 mg/d + ADT	ADT	BCR	60 T: 31 C: 29	T: 67 C: 68.5	T: 33 C: 30	T: 1 (3.4) C: 3 (12)	T: 0 (0) C: 0 (0)
			Apalutamide 240 mg/d monotherapy	ADT		58 T: 29 C: 29	T: 66 C: 68.5	T: 31 C: 30	T: 12 (46) C: 3 (12)	T: 12 (46) C: 0 (0)
Maluf et al (2021) [20]	LACOG 0415	2017–2019	Apalutamide monotherapy	Abiraterone + ADT	Localized PC BCR mHSPC	84 T: 42 C: 42	T: 69.5 C: 69	14	T: 23 (55) C: 3 (7)	T: 6 (14) C: 0 (0)
				Apalutamide + abiraterone		86 T: 42 C: 44	T: 69.5 C: 71	14	T: 23 (55) C: 9 (20)	T: 6 (14) C: 2 (4.5)
Iguchi et al (2020) [21]	OCUU-CRPC/NCT02346578	2015–2018	Enzalutamide 160 mg/d + ADT	Flutamide 375 mg/d + ADT	CRPC	103 T: 52 C: 51	T: 78 C: 77	NA	NA	T: 0 (0) C: 1 (1.9)
*Prophylactic treatment for gynecomastia or breast pain*										
Serretta et al (2012) [11]	NA	2005–2007	Prophylactic TAM 10 mg/d + bicalutamide monotherapy 150 mg/d	Bicalutamide monotherapy 150 mg/d	Localized PCa	163 T: 80 C: 83	T: 73 C: 75	T: 12 C: 24	T: 25 (31) C: 51 (61)	T: 27 (34) C: 48 (58)
Ozen et al (2010) [12]	NA	2003–2005	Prophylactic radiation therapy + bicalutamide monotherapy 150 mg/d	Bicalutamide monotherapy 150 mg/d	Localized PCa	105 T: 44 C: 61	12	12.8	T: 7 (16) C: 31 (51)	T: 16 (36) C: 30 (49)
Bedognetti et al (2010) [13]	NA	2003–2006	Prophylactic TAM 20 mg/d + bicalutamide monotherapy 150 mg/d	Prophylactic TAM 20 mg/d for the first 8 wk + bicalutamide monotherapy 150 mg/d	Localized PCa BCR	80 T: 41 C: 39	T: 65 C: 66	24.2	T: 13 (32) C: 29 (74)	T: 5 (12) C: 18 (46)
Fradet et al (2007) [22]	NA	2002–2003	Prophylactic TAM 20 mg/d + bicalutamide monotherapy 150 mg/d for 12 mo	Placebo + bicalutamide monotherapy 150 mg/d for 12mo	HSPC without metastasis	95 T: 35 C: 60	T: 74 C: 75	12	T: 5 (14) C: 49 (82)	T: 4 (11) C: 58 (97)
			Prophylactic TAM 10 mg/d + bicalutamide monotherapy 150 mg/d for 12 mo			94 T: 34 C: 60	T: 75 C: 75	12	T: 11 (32) C: 49 (82)	T: 10 (29) C: 58 (97)
Saltzstein et al (2005) [23]	NA	NA	Prophylactic TAM 20 mg/d + bicalutamide monotherapy 150 mg/d	Placebo + bicalutamide monotherapy 150 mg/d	HSPC without metastasis	71 T: 35 C: 36	T: 65 C: 67	3	T: 1 (2.8) C: 15 (42)	T: 4 (11) C: 24 (67)
Perdonà et al (2005) [24]	NA	2002–2004	Prophylactic radiation therapy + bicalutamide monotherapy 150 mg/d	Bicalutamide monotherapy 150 mg/d	HSPC without metastasis	101 T: 50 C: 51	T: 71 C: 69	25	T: 17 (34) C: 35 (69)	T: 15 (30) C: 29 (57)
			Prophylactic TAM 10 mg/d + bicalutamide monotherapy 150 mg/d			101 T: 50 C: 51	T: 68 C: 69		T: 4 (8) C: 35 (69)	T: 3 (6) C: 29 (57)
Boccardo et al (2005) [25]	NA	2000–2002	Prophylactic TAM 20 mg/d + bicalutamide monotherapy 150 mg/d	Placebo + bicalutamide monotherapy 150 mg/d	Localized PCa BCR	77 T: 40 C: 37	T: 72 C: 74	NA	T: 4 (10) C: 27 (73)	T: 2 (5) C: 16 (43)
Tyrrell et al (2004) [26]	NA	1999–2001	Prophylactic radiation therapy + bicalutamide monotherapy 150 mg/d	Bicalutamide monotherapy 150 mg/d	HSPC without metastasis	106 T: 52 C: 54	T: 69 C: 70	NA	T: 28 (54) C: 49 (91)	T: 1 (2) C: 5 (9)
Widmark et al (2003) [27]	SPCG-7/SFUO-3	2000	Prophylactic radiation therapy + CAB (leprolin 11.25 mg + flutamide 750 mg)	CAB (leprolin 11.25 mg + flutamide 750 mg)	Localized PCa	253 T: 174 C: 79	NA	12	T: 48 C: 56	T: 111 C: 67

ADT = androgen deprivation therapy; BCR = biochemical recurrence; C = control group; CAB = combined androgen blockade; CRPC = castration-resistant prostate cancer; DTX = docetaxel; HSPC = hormone-sensitive prostate cancer; mHSPC = metastatic hormone-sensitive prostate cancer; NA = not applicable; PCa = prostate cancer; RT = radiotherapy; T = treatment group; TAM = tamoxifen.

### 3.2 Risk of bias assessment

**Fig. 2:**
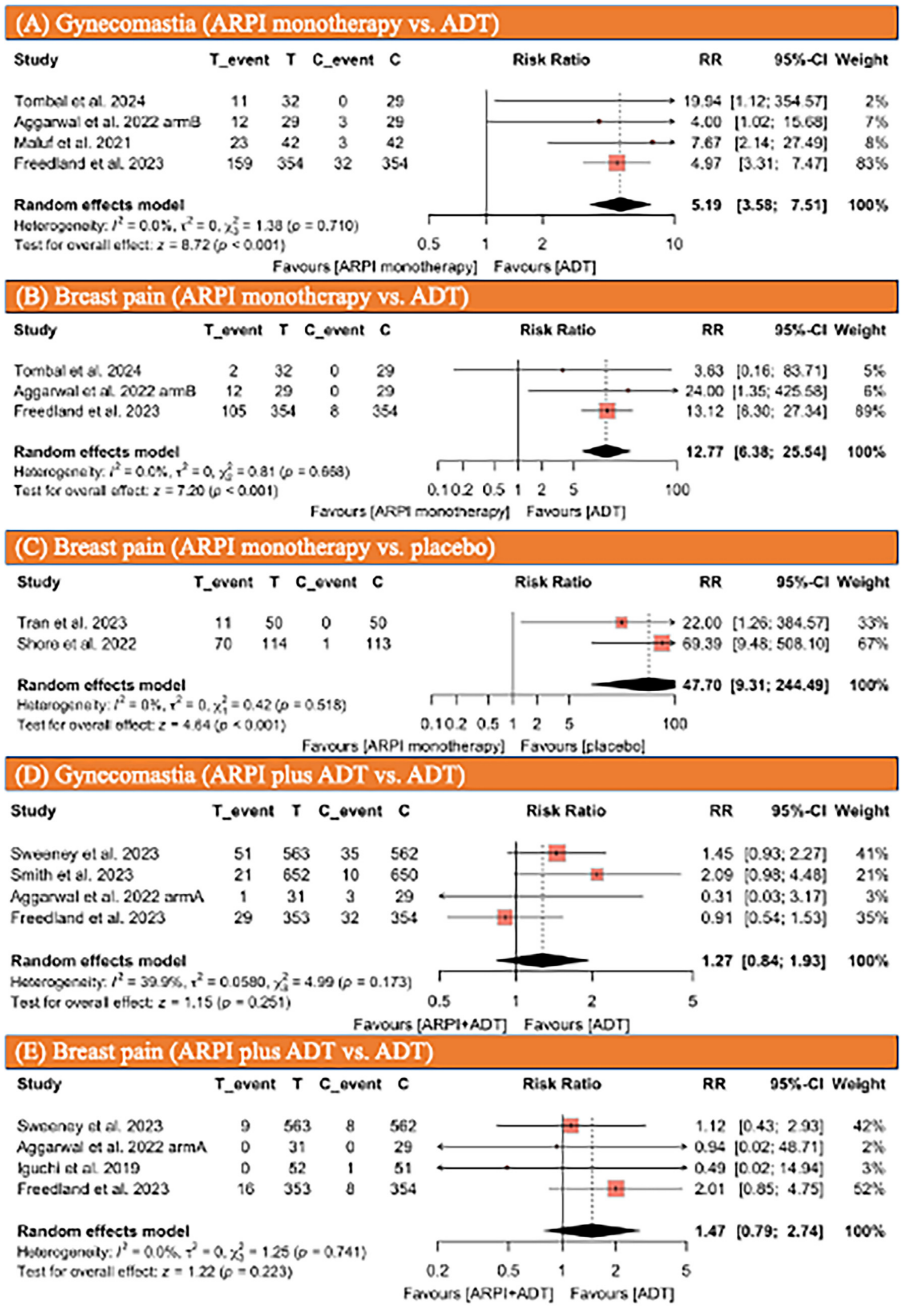
Forest plots illustrating (A) the incidence of gynecomastia in patients receiving APRI monotherapy compared with those on ADT, (B) breast pain in patients receiving APRI monotherapy compared with those on ADT, (C) breast pain in patients receiving APRI monotherapy compared with those on placebo, (D) gynecomastia in patients receiving an APRI in combination with ADT therapy compared with those on ADT alone, and (E) breast pain in patients receiving an APRI in combination with ADT therapy compared with those on ADT alone. ADT = androgen deprivation therapy; ARPI = androgen receptor pathway inhibitor; C = control group; CI = confidence interval; RR = risk ratio; T = treatment group.

**Fig. 3:**
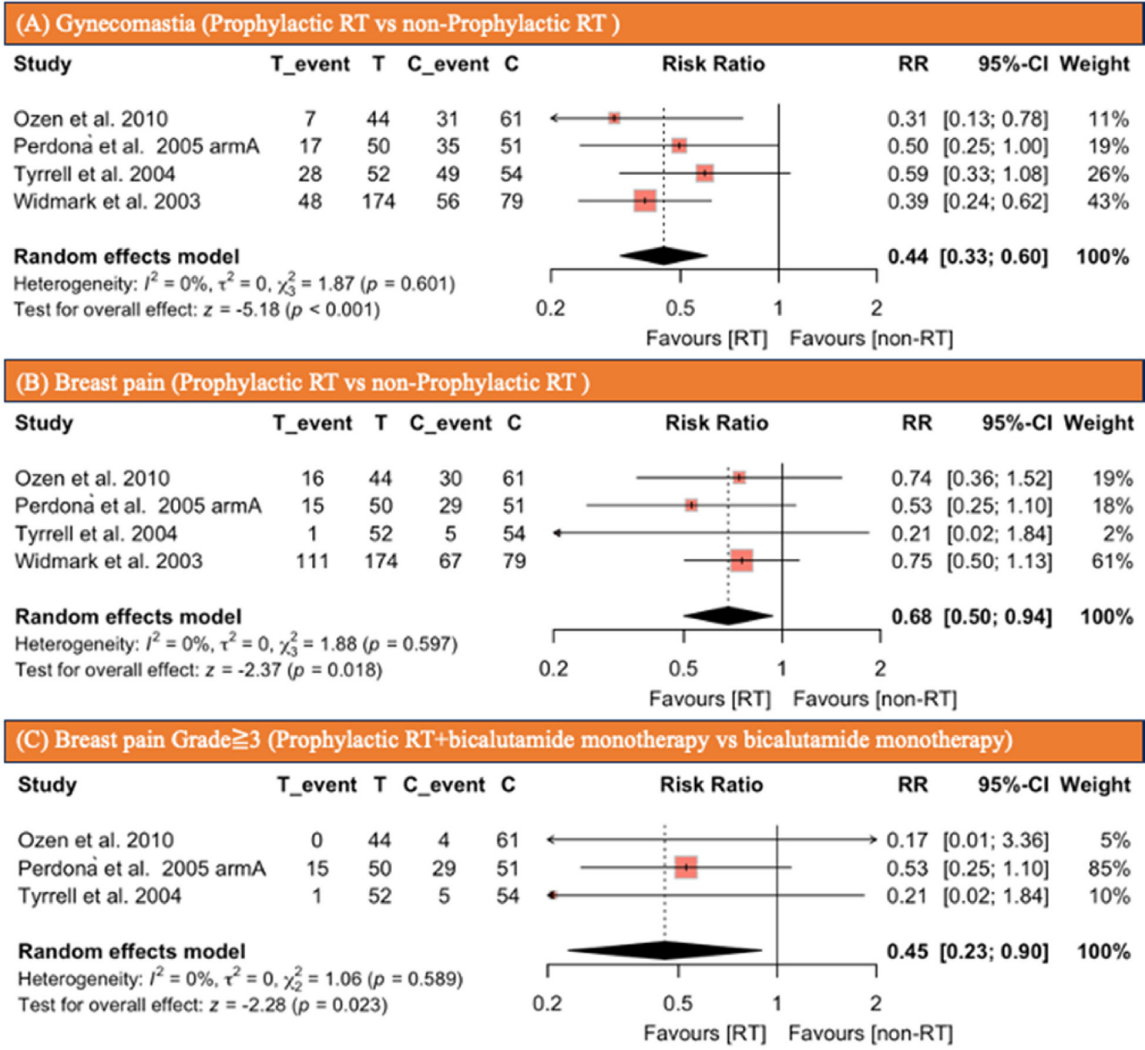
Forest plots illustrating the incidence of (A) gynecomastia, (B) breast pain, and (C) breast pain grade ≥III in patients receiving prophylactic RT compared with not those without prophylactic RT. C = control group; CI = confidence interval; RR = risk ratio; RT = radiotherapy; T = treatment group.

**Fig. 4:**
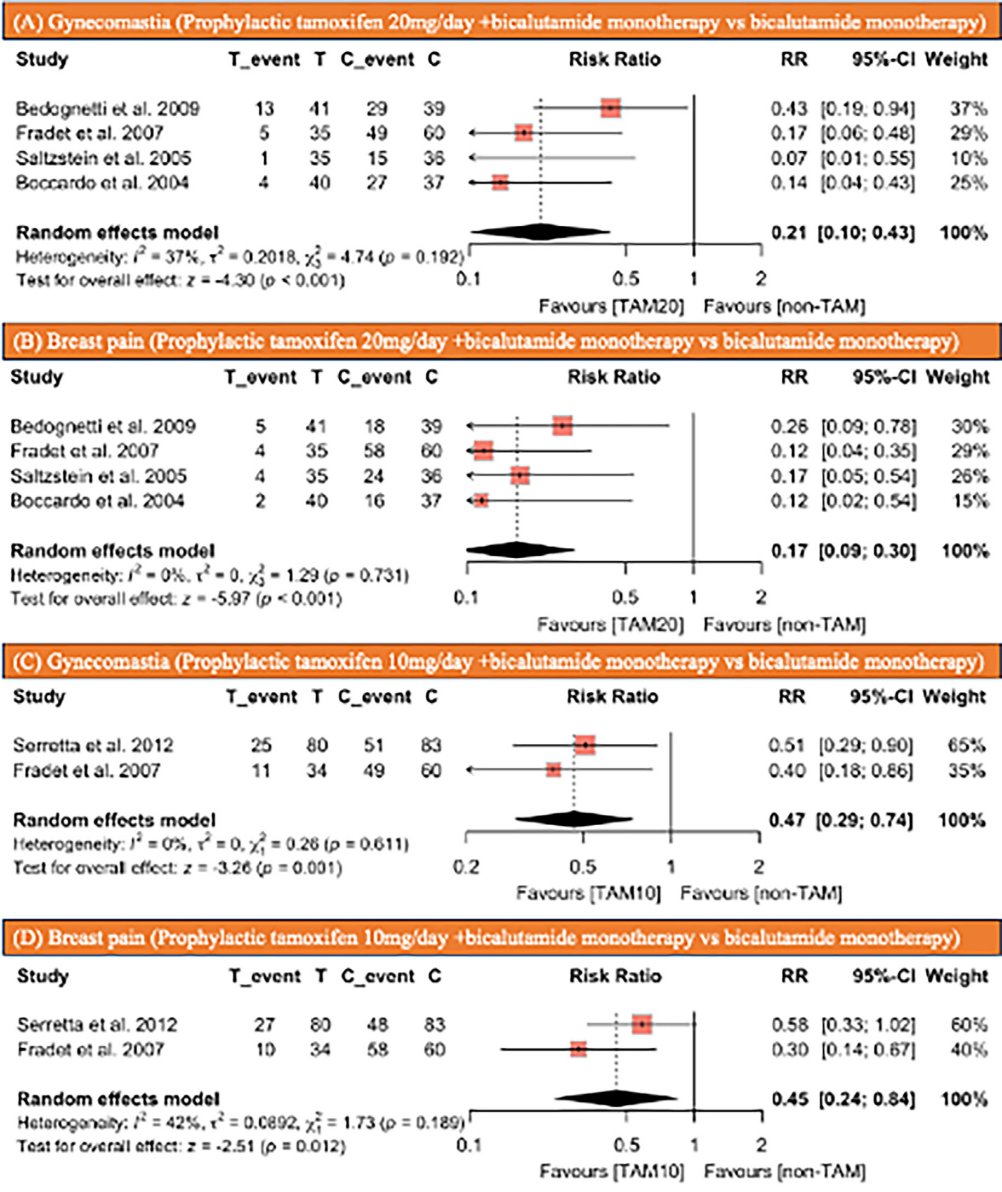
Forest plots illustrating the incidence of (A) gynecomastia and (B) breast pain in patients receiving prophylactic tamoxifen at a dosage of 20 mg/d compared with those without prophylactic tamoxifen, and the incidence of (C) gynecomastia and (D) breast pain in patients receiving prophylactic tamoxifen at a dosage of 10 mg/d compared with those without prophylactic tamoxifen. C = control group; CI = confidence interval; RR = risk ratio; T = treatment group; TAM = tamoxifen.

The judgment of the authors regarding each domain for each included study is presented in Supplementary Fig. 2. Forrest plot and funnel plots of each analysis are depicted in Fig. 2, Fig. 3, Fig. 4, and Supplementary Fig. 1.

#### 3.2.1 Incidence of gynecomastia and breast pain in PCa patients treated with ARPI therapy

##### 3.2.1.1 ARPI monotherapy versus ADT monotherapy

Four studies [2], [5], [19], [20], comprising 911 patients, reported the incidence of gynecomastia in PCa patients treated with ARPI monotherapy compared with those treated with ADT monotherapy. The studies included 457 patients in the ARPI monotherapy group and 454 patients in the ADT monotherapy group. In the ARPI monotherapy group, 205 patients (45%) experienced gynecomastia, compared with 38 patients (8.4%) in the ADT monotherapy group. The patients who received ARPI monotherapy had a significantly higher incidence of gynecomastia than those who received ADT monotherapy (RR: 5.19, 95% CI: 3.58–7.51, *p* < 0.001; Fig. 2A). No significant heterogeneity was observed according to the Cochran’s Q tests and *I^2^* statistic.

Three studies [2], [5], [19], comprising 827 patients, reported the incidence of breast pain in PCa patients treated with ARPI monotherapy compared with those treated with ADT monotherapy. The studies included 415 patients in the ARPI monotherapy group and 412 patients in the ADT monotherapy group. In the ARPI monotherapy group, 119 patients (29%) experienced breast pain, compared with eight patients (2%) in the ADT monotherapy group. The patients who received ARPI monotherapy had a significantly higher incidence of breast pain than those who received ADT monotherapy (RR: 12.77, 95% CI: 6.38–25.54, *p* < 0.001; Fig. 2B). No significant heterogeneity was observed according to the Cochran’s Q tests and *I^2^* statistic.

Two studies [3], [7], comprising 327 patients, reported the incidence of breast pain in PCa patients treated with ARPI monotherapy compared with placebo. The studies included 164 patients in the ARPI monotherapy group and 163 patients in the ADT monotherapy group. In the ARPI monotherapy group, 81 patients (49%) experienced breast pain, compared with one patient (0.6%) in the ADT monotherapy group. The patients who received ARPI monotherapy had a significantly higher incidence of breast pain than those who received placebo (RR: 47.7, 95% CI: 9.31–244.49; Fig. 2C). No significant heterogeneity was observed according to the Cochran’s Q tests and *I^2^* statistic.

##### 3.2.1.2 ARPI plus ADT versus ADT monotherapy

Four studies [4], [5], [6], [19], comprising 3204 patients, reported the incidence of gynecomastia, and four studies [4], [5], [19], [21], comprising 1995 patients, reported the incidence of breast pain in PCa patients treated with ARPI plus ADT therapy compared with those treated with ADT monotherapy. Regarding gynecomastia, the studies included 1599 patients in the ARPI plus ADT therapy group and 1605 patients in the ADT monotherapy group. In the ARPI plus ADT therapy group, 102 patients (6.4%) experienced gynecomastia, compared with 80 patients (5%) in the ADT monotherapy group. There was no significant difference in the incidence of gynecomastia between the two groups (RR: 1.27, 95% CI: 0.84–1.93, *p* = 0.2; Fig. 2D). No significant heterogeneity was observed according to the Cochran’s Q tests and *I^2^* statistic.

Regarding breast pain, the studies included 999 patients in the ARPI plus ADT therapy group and 996 patients in the ADT monotherapy group. In the ARPI plus ADT therapy group, 25 patients (2.5%) experienced breast pain, compared with 17 (1.7%) in the ADT monotherapy group. There was no significant difference in the incidence of breast pain between the two groups (RR: 1.47, 95% CI: 0.79–2.74, *p* = 0.2; Fig. 2E). No significant heterogeneity was observed according to the Cochran’s Q tests and *I^2^* statistic.

#### 3.2.2 Efficacy of prophylactic treatment for gynecomastia and breast pain caused by antiandrogen therapy

##### 3.2.2.1 Prophylactic RT

Four studies [12], [24], [26], [27], comprising 565 patients, reported the efficacy of prophylactic RT for gynecomastia and breast pain due to antiandrogen therapy. Three studies [12], [24], [26] used bicalutamide monotherapy, and one study [27] used combined androgen blockade (leprolin plus flutamide). The studies included 320 patients undergoing antiandrogen therapy with prophylactic RT and 245 patients undergoing antiandrogen therapy without prophylactic RT. In the prophylactic RT group, 100 patients (31%) experienced gynecomastia, compared with 171 (70%) in the nonprophylactic RT group. The incidence of gynecomastia was significantly lower in patients who underwent prophylactic RT than in those who did not (RR: 0.44, 95% CI: 0.33–0.6, *p* < 0.001; Fig. 3A). In the prophylactic RT group, 143 patients (45%) experienced breast pain, compared with 131 (53%) in the group that did not receive prophylactic RT. The patients who underwent prophylactic RT had a significantly lower incidence of breast pain than those who did not (RR: 0.68, 95% CI: 0.5–0.94, *p* = 0.018; Fig. 3B). No significant heterogeneity was observed according to the Cochran’s Q tests and *I^2^* statistic.

Three studies [12], [24], [26], comprising 312 patients, reported the efficacy of prophylactic RT for breast pain more than grade III due to antiandrogen therapy. The studies included 146 patients undergoing antiandrogen therapy with prophylactic RT and 166 patients undergoing antiandrogen therapy without prophylactic RT. Sixteen patients (11%) experienced breast pain of grade ≥III in the prophylactic RT group, compared with 38 (23%) in the nonprophylactic RT group. The patients who underwent prophylactic RT had a significantly lower incidence of breast pain of grade ≥III than those who did not (RR: 0.45, 95% CI: 0.23–0.90, *p* = 0.023; Fig. 3C). No significant heterogeneity was observed according to the Cochran’s Q tests and *I^2^* statistic.

##### 3.2.2.2 Prophylactic tamoxifen

Four studies [13], [22], [23], [25], comprising 323 patients, reported the efficacy of prophylactic tamoxifen 20 mg/d for gynecomastia and breast pain due to bicalutamide monotherapy. The studies included 151 patients receiving antiandrogen therapy with prophylactic tamoxifen 20 mg/d and 172 patients receiving antiandrogen therapy without prophylactic tamoxifen 20 mg/d. In the group receiving prophylactic tamoxifen at a dose of 20 mg/d, 23 patients (15%) experienced gynecomastia, compared with 120 (70%) in the nonprophylactic tamoxifen group. The patients who received prophylactic tamoxifen at a daily dose of 20 mg had a significantly lower incidence of gynecomastia than those who did not (RR: 0.21, 95% CI: 0.1–0.43, *p* < 0.001; Fig. 4A). In the group receiving prophylactic tamoxifen at a dose of 20 mg/d, 15 patients (10%) experienced breast pain, compared with 140 (81%) in the nonprophylactic tamoxifen group. The patients who received prophylactic tamoxifen at a daily dose of 20 mg had a significantly lower incidence of breast pain than those who did not (RR: 0.17, 95% CI: 0.09–0.30, *p* < 0.001; Fig. 4B).

Two studies [11], [22], comprising 257 patients, reported the efficacy of prophylactic tamoxifen 10 mg/d for gynecomastia and breast pain due to bicalutamide monotherapy. The studies included 114 patients receiving antiandrogen therapy with prophylactic tamoxifen 10 mg/d and 143 patients receiving antiandrogen therapy without prophylactic tamoxifen. In the group receiving prophylactic tamoxifen 10 mg/d, 36 patients (32%) experienced gynecomastia, compared with 100 (70%) in the group not receiving prophylactic tamoxifen. The patients who received prophylactic tamoxifen at a daily dose of 10 mg had a significantly lower incidence of gynecomastia than those who did not (RR: 0.47, 95% CI: 0.29–0.74, *p* = 0.001; Fig. 4C). In the prophylactic tamoxifen 10 mg/d group, 37 patients (32%) experienced breast pain, compared with 106 (74%) in the nonprophylactic tamoxifen group. The patients who received prophylactic tamoxifen at a daily dose of 10 mg had a significantly lower incidence of breast pain than those who did not (RR: 0.45, 95% CI: 0.24–0.84, *p* = 0.012; Fig. 4D).

## 4 Discussion

This systematic review and meta-analysis of RCTs aims to analyze the incidence of gynecomastia and breast pain due to ARPIs compared with ADT and the effect of prophylactic tamoxifen and RT in patients who received bicalutamide monotherapy. Our study reveals several findings. First, patients who received ARPI monotherapy exhibited significantly higher incidences of both gynecomastia and breast pain than those who received ADT monotherapy. Second, there was no significant difference in the incidence of gynecomastia and breast pain between patients receiving ARPI plus ADT therapy and those receiving ADT monotherapy. Third, either prophylactic tamoxifen or RT significantly decreased the incidence of gynecomastia and breast pain due to bicalutamide monotherapy. We could not find the research regarding the effect of prophylactic treatment for gynecomastia and breast pain caused by ARPIs.

We found that ARPI monotherapy is associated with approximately fivefold increase in gynecomastia and a 13-fold increase in breast pain compared with ADT monotherapy. Bicalutamide monotherapy results in an increase in serum testosterone levels. The increased testosterone is converted into estradiol by the enzyme aromatase in peripheral tissues. The subsequent elevation in estradiol levels is postulated to induce the enlargement of breast gland tissue, causing breast tenderness [10]. Previous studies [2], [20] revealed that ARPI monotherapy, including enzalutamide and darolutamide, also increased serum testosterone levels after 1 mo. It is considered that gynecomastia and breast pain may result from the same mechanism, as well as bicalutamide. Thus, when ARPI is used in conjunction with luteinizing hormone-releasing hormone agonists or gonadotropin-releasing hormone antagonists, the incidence of gynecomastia may be expected to be reduced. The results of our study supported this hypothesis, by indicating that an ARPI added to ADT therapy did not increase the incidence of gynecomastia and breast pain compared with ADT monotherapy. Previous RCTs [28], [29], [30] have demonstrated that ARPI plus ADT therapy is a standard treatment for patients with hormone-sensitive and castration-resistant PCa. In these cases, it is not necessary to give as much consideration to gynecomastia or breast pain as in cases where ARPI monotherapy is used.

In our analysis regarding the incidence of gynecomastia and breast pain due to ARPI monotherapy, approximately half of the patients exhibited gynecomastia and approximately one-third suffered from breast pain. Although we could not compare the incidence between bicalutamide monotherapy and ARPI monotherapy, given that the incidence of gynecomastia and breast pain due to bicalutamide monotherapy was 70% [31], it can be concluded that ARPI monotherapy is slightly better than bicalutamide monotherapy. However, the occurrence of these AEs in patients receiving ARPI monotherapy necessitates the consideration of appropriate treatment strategies. Unfortunately, no studies were identified that investigated the effect of prophylactic treatment for gynecomastia due to ARPI monotherapy. Our study demonstrated that either prophylactic tamoxifen administration or RT significantly reduced the incidence of gynecomastia and breast pain due to bicalutamide monotherapy. Specifically, we revealed that prophylactic RT was associated with a significantly lower incidence of grade ≥III breast pain. If the underlying mechanisms of gynecomastia and breast pain due to ARPI monotherapy are analogous to those observed with bicalutamide monotherapy, then it can be expected that these preventative strategies will be effective.

Only one study [24] reported the effect of prophylactic treatment for gynecomastia comparing tamoxifen and RT. The incidence of grade ≥III gynecomastia was 8% and 34%, respectively. Regarding to the AEs associated with the prophylactic treatment, patients who received RT experienced a significantly higher incidence of rash, nipple erythema, or skin irritation than those who received tamoxifen (2% vs 38%, *p* = 0.01). Furthermore, it also revealed that tamoxifen, when used as a symptomatic treatment for gynecomastia caused by bicalutamide monotherapy, reduced the frequency of gynecomastia significantly compared with RT (odds ratio: 0.2, 95% CI: 0.18–0.22, *p* = 0.02). Tamoxifen may be more effective than RT in the prevention and treatment of gynecomastia.

One study [13] investigated the differentiation in the effect of prophylactic tamoxifen based on the dosage. The patients were divided into five groups according to the dosage of tamoxifen: 1, 2.5, 5, 10, and 20 mg. Prophylactic administration of tamoxifen at a dosage of 20 mg was most effective in preventing gynecomastia at 12 mo among all dosage groups (placebo: 80%, 1 mg: 79%, 2.5 mg: 47%, 5 mg: 32%, 10 mg: 24%, and 20 mg: 6%). Patients treated with tamoxifen experienced hot flushes most frequently. The study showed that the incidence of hot flushes was higher in patients receiving tamoxifen doses exceeding 5 mg/d. We believe that it is important to inform patients about the dose-dependent effect and elevated risk of hot flushes associated with prophylactic tamoxifen use.

### 4.1 Limitations

This study is subject to certain limitations. Primarily, several studies included only a small number of patients. Second, it was not possible to perform a subgroup analysis according to the ARPI type or the timing of ARPI usage. Moreover, we could not find the data regarding the incidence of breast pain or gynecomastia in some essential RCTs, such as SPARTAN, ARAMIS trials, and so on. If we could find and extract the data from these important trials, subgroup analyses based on the type of ARPIs may have been possible. Third, we used RR as a following reason. A previous study [32] suggested that gynecomastia and breast pain occur most frequently within 6–9 mo after the initiation of bicalutamide monotherapy. As we described, it is likely that a similar mechanism is involved in gynecomastia due to ARPI monotherapy. Given that the median follow-up duration of the included studies was >1 yr, we thought that RR could be used instead of person-years at risk. Moreover, we think the these differences, such as the length of follow-up duration and type of antiandrogen medication, create heterogeneity in our analyses, although no significant heterogeneity was observed. Fourth, all studies examining prophylactic treatment used bicalutamide as antiandrogen monotherapy. To the best of our knowledge, there have been no studies investigating the effect of prophylactic treatment of gynecomastia during ARPI monotherapy.

## 5 Conclusions

We found that patients who received ARPI monotherapy had a significantly higher incidence of gynecomastia and breast pain than those receiving ADT monotherapy. On the contrary, there was no significant difference in the incidence of gynecomastia and breast pain between ARPI plus ADT therapy and ADT monotherapy. The administration of prophylactic tamoxifen or RT was found to significantly reduce the incidence of both gynecomastia and breast pain caused by bicalutamide monotherapy. In conclusion, we suggest that prophylactic treatment may be considered when administering ARPI monotherapy to patients with PCa, as the number of patients experiencing AEs due to ARPI monotherapy is expected to rise. Further studies are needed to clarify the effect of prophylactic treatment for gynecomastia and breast pain in the setting of ARPI monotherapy.

***Author contributions*:** Shahrokh F. Shariat had full access to all the data in the study and takes responsibility for the integrity of the data and the accuracy of the data analysis.

*Study concept and design*: Tsuboi, Schulz.

*Acquisition of data*: Tsuboi.

*Analysis and interpretation of data*: Tsuboi.

*Drafting of the manuscript*: Tsuboi, Schulz.

*Critical revision of the manuscript for important intellectual content*: Laukhtina, Wada, Karakiewicz, Araki, Shariat.

*Statistical analysis*: Tsuboi.

*Obtaining funding*: None.

*Administrative, technical, or material support*: None.

*Supervision*: Araki, Shariat.

*Other*: None.

***Financial disclosures:*** Shahrokh F. Shariat certifies that all conflicts of interest, including specific financial interests and relationships and affiliations relevant to the subject matter or materials discussed in the manuscript (eg, employment/affiliation, grants or funding, consultancies, honoraria, stock ownership or options, expert testimony, royalties, or patents filed, received, or pending), are the following: Shahrokh F. Shariat received honoraria from Astellas, AstraZeneca, BMS, Ferring, Ipsen, Janssen, MSD, Olympus, Pfizer, Roche, and Takeda; reports a consulting or advisory role at Astellas, AstraZeneca, BMS, Ferring, Ipsen, Janssen, MSD, Olympus, Pfizer, Pierre Fabre, Roche, and Takeda; and reports being in the speakers’ bureaus of Astellas, Astra Zeneca, Bayer, BMS, Ferring, Ipsen, Janssen, MSD, Olympus, Pfizer, Richard Wolf, Roche, and Takeda.

***Funding/Support and role of the sponsor*:** None.
